# Printable hybrid hydrogel by dual enzymatic polymerization with superactivity†
†Electronic supplementary information (ESI) available: Experimental details and supplementary figures. See DOI: 10.1039/c5sc02234g


**DOI:** 10.1039/c5sc02234g

**Published:** 2016-01-04

**Authors:** Qingcong Wei, Mengchi Xu, Chuanan Liao, Qing Wu, Mingyu Liu, Ye Zhang, Chengtie Wu, Liming Cheng, Qigang Wang

**Affiliations:** a Department of Chemistry , Advanced Research Institute , Tongji University , Shanghai 200092 , P. R. China . Email: wangqg66@tongji.edu.cn; b State Key Laboratory of Performance Ceramics and Superfine Microstructure , Shanghai Institute of Ceramics , Chinese Academy of Sciences , Shanghai 200050 , P. R. China . Email: chengtiewu@mail.sic.ac.cn; c School of Life Sciences and Technology , Tongji University , Shanghai 200092 , P. R. China; d Bioinspired Soft Matter Unit , Okinawa Institute of Science and Technology , Okinawa , Japan . Email: ye.zhang@oist.jp; e Spine Division of Orthopaedics Department , Tongji Hospital , Tongji University School of Medicine , Shanghai 200065 , P. R. China

## Abstract

A new approach has been developed to fabricate tough hybrid hydrogels by employing dual enzyme-mediated redox initiation to achieve post-self-assembly cross-linking polymerization. The resulting hydrogel combines the merits of supramolecular hydrogels with polymeric hydrogels to achieve higher mechanical strength and porous networks. Designed 3D constructs were fabricated *via in situ* 3D printing. The *in situ* immobilized GOx/HRP in Gel II exhibited superactivity compared to free enzymes, which might be attributed to the synergistic effect of co-localized GOx and HRP minimizing the distances for mass transport between the gel and the bulk solution. This mechanically strong hybrid hydrogel maintained high reusability and thermal stability as well. In addition, *in situ* 3D cell culture was demonstrated, thus indicating that this biodegradable hybrid hydrogel is biocompatible with cells. The subsequent 3D cell printing further indicates that the hybrid hydrogel is a promising scaffold for bio-related applications such as biocatalysis and tissue engineering.

## Abstract 2

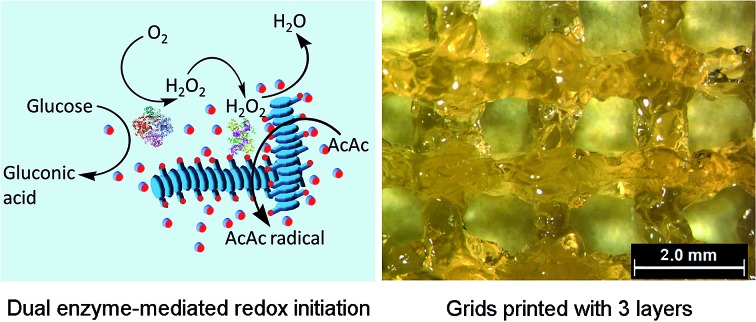
Hybrid hydrogels were fabricated via a new approach employing a dual enzyme-mediated redox initiation reaction and their applications for 3D printing and biocatalysis.

## Introduction

Hydrogels with three-dimensional (3D) fibril networks have received extensive attention due to their successful applications in drug delivery,^1^ tissue engineering,^2^ biocatalysis,^3^–^5^ cell culture,^6^ selective adsorption,^7^,^8^ wound healing^9^ and 3D printing.^10^–^15^ In general, supramolecular hydrogels formed *via* small peptides nanofibers^16^–^18^ are suitable scaffolds for the entrapment of biomolecules and living cells. However, they normally have poor elasticity and weak mechanical properties. Covalently cross-linked networks (polymeric hydrogel)^2^ can deform elastically and still maintain strong mechanical properties.^19^ However, they normally lack a suitable porous structure for the diffusion of substrates with large sizes, which is an essential factor for cell cultures and biocatalysis.

One effective approach to secure both advantages is combining supramolecular hydrogels with polymeric materials.^20^ Smith *et al.* prepared robust hybrid hydrogels *via* the assembly of a hydrogelator within polysaccharide hydrogel networks.^21^ Yang *et al.* and Adams *et al.* also showed that mixing supramolecular hydrogels with agarose and dextran leads to novel hybrid hydrogels with tunable mechanical strength.^22^,^23^ Recently, our group developed polymer enhanced hybrid self-recovering hydrogels *via* glucose oxidase mediated polymerization.^24^ Another post-self-assembly cross-linking approach was reported by Xu *et al.*, who demonstrated that *in situ* photo-polymerization of acrylic modified oligopeptide hydrogelators with copolymers can achieve a tough hydrogel.^25^,^26^ Moreover, these hydrogels have been utilized in multiple applications such as the immobilization of enzymes, controlled release of drugs, controlled cell adhesion and biomimetic materials.^21^–^24^,^27^–^29^

Despite the success in designing hybrid hydrogels, the application of 3D printing to the fabrication of tough hybrid hydrogels is still a big challenge for generating complex tissues. Therefore, we report herein a dual enzyme-mediated redox initiation to achieve post-self-assembly cross-linking of acrylic modified hydrogelators (NapFFK-acrylic acid, Scheme S1a†)^25^,^26^ with monomers for hybrid hydrogel generation. The injectable supramolecular hydrogel is printed into a 3D structure due to the sol–gel transition and quick recovery during the pressure-driven prototyping. The further reinforced process initiated by the dual enzymatic polymerization can gradually increase the toughness of the hydrogel without any additional curing process.

**Fig. 1:**
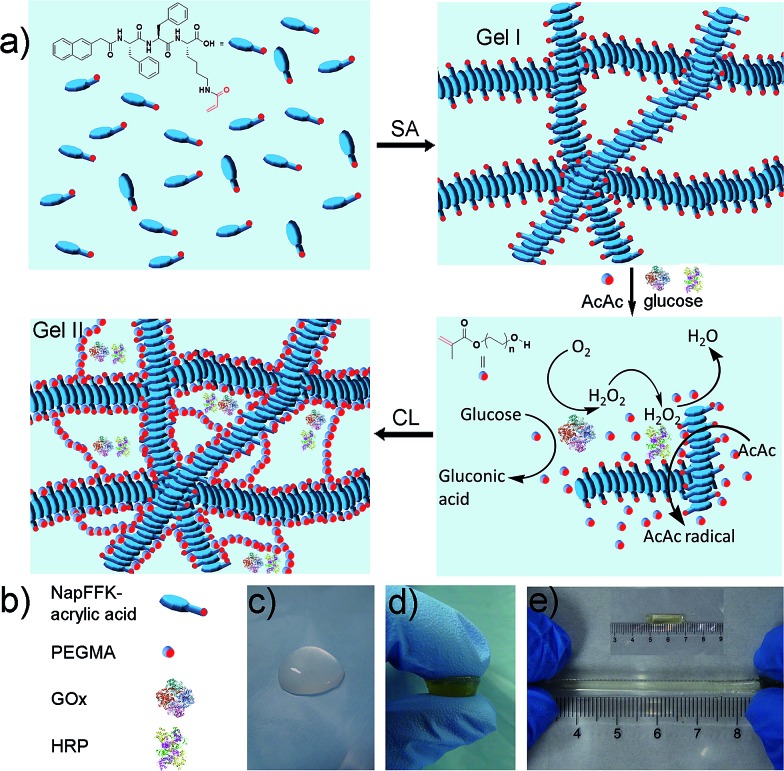
(a) Schematic of the preparation of the hybrid hydrogel, Gel II (SA = self-assembly, CL = cross-linking). (b) Schematic of NapFFK-acrylic acid, PEGMA, GOx and HRP. (c) Optical image of Gel I. (d) Optical image of Gel II under compression. (e) The tensile state of Gel II.

There are two steps (Fig. 1a) in the preparation of the hybrid hydrogel: (i) self-assembly (Gel I) and (ii) polymerization/cross-linking (Gel II). The selected dual enzyme system is composed of glucose oxidase (GOx), glucose, horseradish peroxidase (HRP), acetyl acetone (AcAc) and poly(ethylene glycol) methacrylate (PEGMA, Scheme S1b†), which integrates a GOx-mediated redox system with an HRP-mediated redox system.^30^–^34^ Initially, the GOx catalyzes the oxidation of glucose to gluconic acid. Subsequently, the flavin adenine dinucleotide (FAD) cofactor reduces O_2_ to H_2_O_2_. Then, AcAc radicals generated *via* the HRP-catalyzed oxidation of AcAc with H_2_O_2_ initiate the polymerization of PEGMA with acrylic modified hydrogelators. The carbon radical derived from the AcAc molecule was then detected by electron paramagnetic resonance (EPR) measurements (Fig. S1†).

## Results and discussion

The preparation of a hybrid hydrogel is straightforward. First, a typical supramolecular hydrogel (Gel I, Fig. 1c) was formed *via* pH triggered molecular self-assembly in water. Then, dual enzyme-catalyzed reagents, which include glucose (aq) 50 μL (40 mM), HRP (aq) 10 μL (42 mg mL^–1^), AcAc 10 μL and PEGMA (MW = 360 g mol^–1^) 25 μL, were added to Gel I (325 μL, 1.54 wt% hydrogelator), then GOx(aq) 100 μL (10 mg mL^–1^) was added. The precursor solution was mixed thoroughly and kept at room temperature to form the hybrid hydrogel, Gel II (Fig. 1d).

The gelation process was monitored *via*^1^H NMR (25 °C). After 120 min, PEGMA vinyl double groups were saturated quantitatively (Fig. S2†), which corresponded to the formation of Gel II, whereas the gelator conversion test showed that 75.3% ± 2.8% (mean ± SD) of NapFFK-acrylic acid participated in the polymerization reaction. In addition, time sweep measurements conducted *via* a rheometer (Fig. S3†), show that a crossover point between the storage modulus (*G*′) and loss modulus (*G*′′) appears at *ca.* 13.5 min (810 s). To choose a rational quantity of PEGMA for use in the cross-linking process, a series of hybrid hydrogels containing different amounts of PEGMA with 0.96 wt% of NapFFK-acrylic acid were prepared for a comparison of mechanical properties. Table S1† elucidates the results of frequency-dependent sweep measurements at a constant strain of 0.03%. The values for both storage modulus (*G*′) and loss modulus (*G*′′) increased gradually with an increase in the amount of PEGMA, whereas there was just a slight increase when the concentration was higher than 4.8 wt%, thus we chose this amount of PEGMA in the following experiments.

**Fig. 2:**
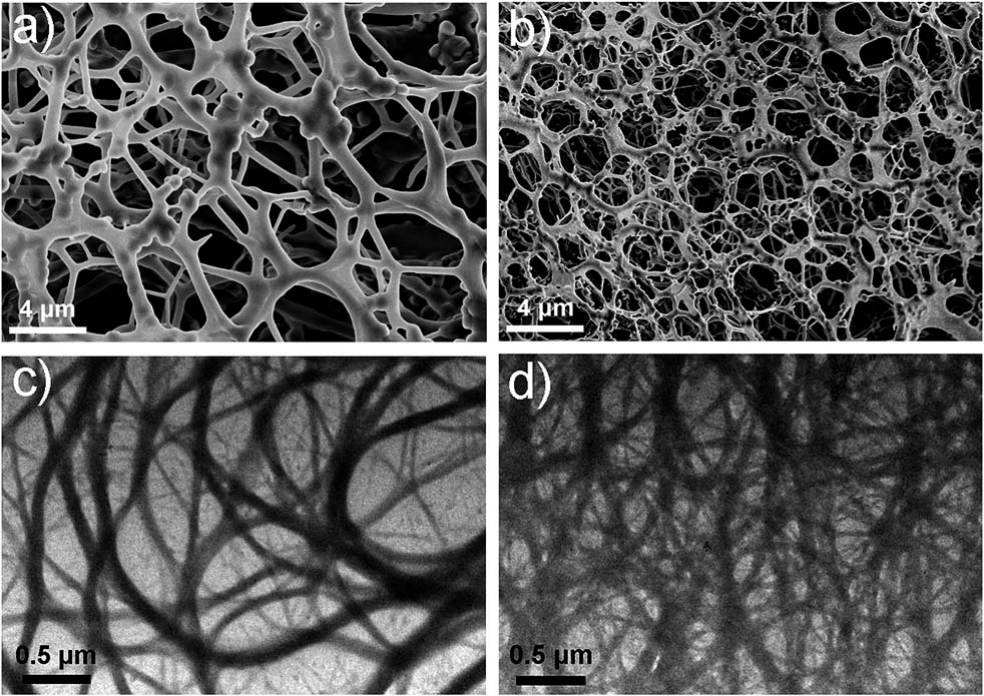
SEM images of Gel I (a) and Gel II (b). TEM images of Gel I (c) and Gel II (d).

**Fig. 3:**
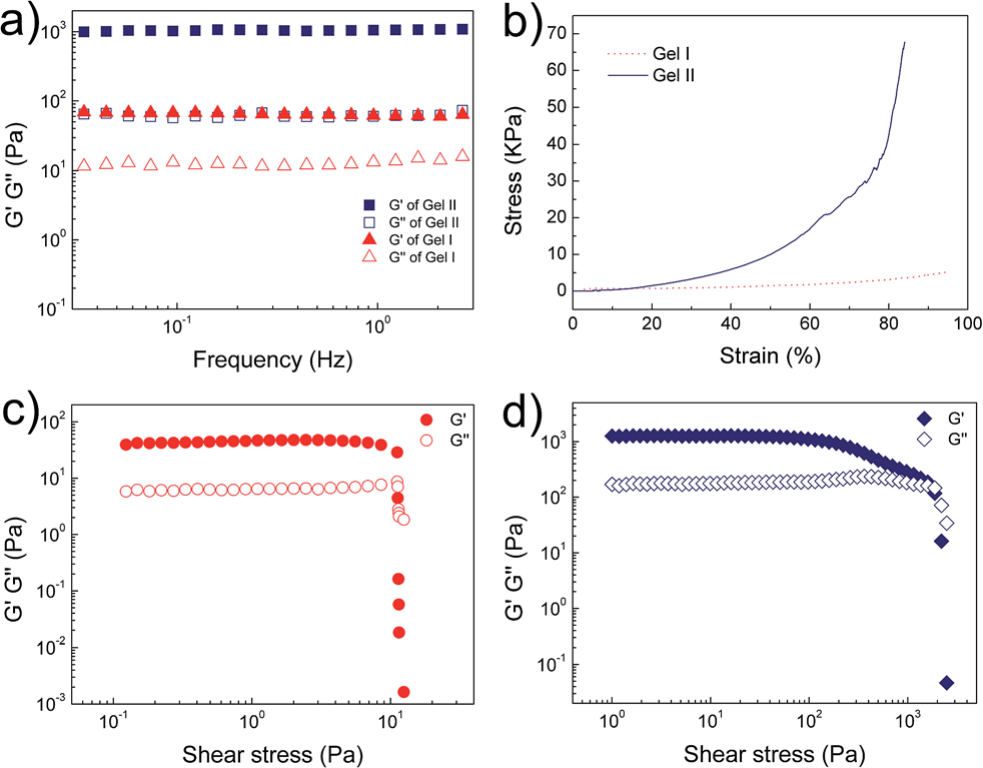
(a) Frequency sweep tests of Gel I and Gel II. (b) Compressive tests of Gel I and Gel II. Oscillatory stress sweep tests of (c) Gel I and (d) Gel II.

Electron microscopy (Fig. 2a–d) confirmed that Gel II had a similar porous network structure as Gel I, but with a relatively smaller pore size. The scanning electron microscopy (SEM) image of Gel I (Fig. 2a) reveals entangled irregular fibers, which comprise the matrix of the supramolecular hydrogel. Gel II had a different morphology with fibers that were tightly cross-linked to each other (Fig. 2b). Although the widths of the nanofibers are similar for both Gel I and Gel II, transmission electron microscopy (TEM) images demonstrate that the density of the nanofibers in Gel II was much higher than the Gel I network, which verifies that the polymerization/cross-linking process facilitates the formation of a polymer bridge between the nanofibers in Gel II. To test the mechanical properties of the gels, Fig. 3a demonstrates the frequency-dependent sweep measurements at a constant strain of 0.03%. The values for storage modulus (*G*′) dominate the loss modulus (*G*′′) for both Gel I and Gel II, which confirm their inherent high elastic properties. The storage modulus (*G*′) of Gel II (*ca.* 1065 Pa) is approximately 16.4 times that of Gel I (*ca.* 65 Pa), which is consistent with the SEM results. In addition, we replaced NapFFK-acrylic acid with NapFFK to prepare a hybrid hydrogel, which has a lower storage modulus (*ca.* 345 Pa) due to the lack of a cross-linking point.

**Fig. 4:**
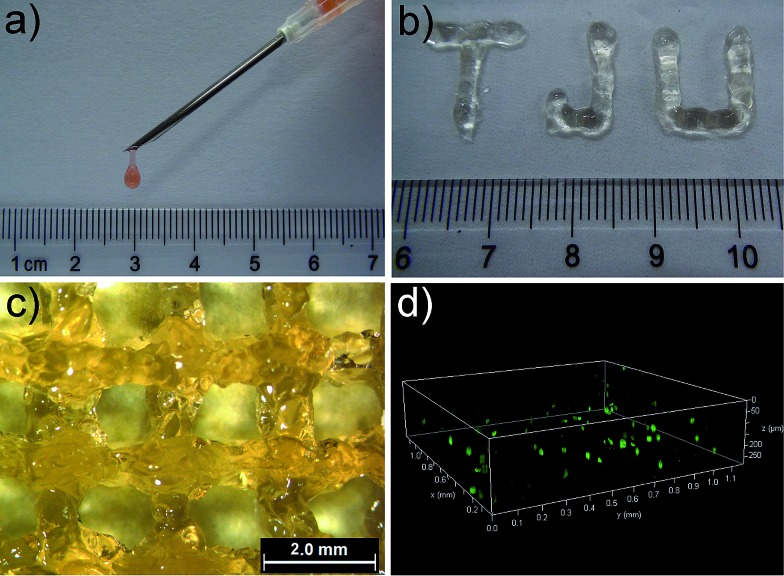
(a) The injectable property of Gel II (stained with Congo red). (b) The printed letters “TJU” with three layers. (c) Grids printed with three layers. (d) The printed 3D stacks of NIH-3T3 cells with FDA staining in green and PI staining in red (gridlines for *x* and *y* axis: 0.1 mm; gridlines for *z* axis: 50 μm).

Gel II can retain its shape even under compression and tensile relative to the weaker Gel I. As shown in Fig. 3b, Gel II can resist over 80% compression and has a 7.39 kPa Young's modulus, whereas Gel I cannot endure compression. Furthermore, the cylindrical (with a diameter of 4.5 mm) hybrid hydrogel could be stretched to about 2.5 times its initial length without collapse (Fig. 1e). The higher yield stress of Gel II (*ca.* 160 Pa) compared to Gel I (*ca.* 11 Pa) also indicates its superior self-standing capability (Fig. 3c and d). In addition, Gel II rapidly recovers its mechanical properties after a large-amplitude oscillatory breakdown (Fig. S4†). Because of these properties (such as *in situ* formation and enhanced mechanical properties of Gel II), we used the precursor solution for 3D printing. As shown in Fig. 4a, the precursor solution of Gel II could be injected *via* a syringe. After mixing thoroughly, the precursor solution was maintained at 37 °C for 5 min and Gel I was formed, and then designed 3D structures could be fabricated in the programmed position on a valve-based printer. The letters “TJU” (Fig. 4b) and grids (Fig. 4c) with three layers were 3D printed. This result demonstrates that the hybrid hydrogels could be used as novel printable scaffold materials with enhanced mechanical properties.

**Fig. 5:**
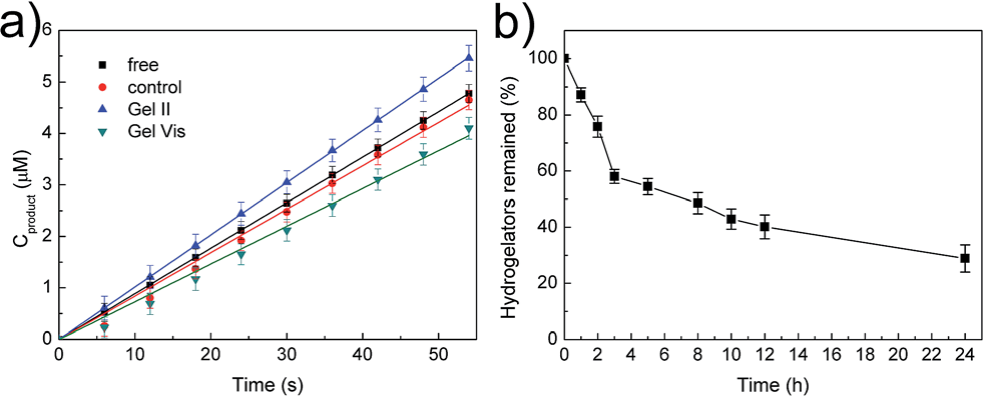
(a) Initial reaction course of *o*-phenylenediamine (OPD) (5 mM) and glucose (5 mM) in buffer catalyzed by various enzymatic systems. (b) Digestion curve of NapFFK-acrylic acid hydrogelators by the treatment of proteinase K.

The tough supramolecular-polymeric hydrogel is an appropriate scaffold for biomolecules. It should be noted that during the polymerization/cross-linking process, the dual enzymes (GOx/HRP) not only generate radicals in the initiation stage,^33^ but also immobilize them into the hybrid hydrogel matrix *in situ*. To explore the catalytic activity of free enzymes *versus* immobilized enzymes, the oxidation of *o*-phenylenediamine (OPD) by H_2_O_2_ was selected as a model reaction with glucose and OPD as substrates.^33^,^34^ Regardless of whether the enzymes were immobilized or not, all tests concerning catalysis were performed with identical concentrations of GOx and HRP (GOx, 5 mg L^–1^; HRP, 2.1 mg L^–1^). Two other systems were prepared with the same protocol as Gel II with two variations: (i) the high concentration supramolecular hydrogel was substituted with the same weight of water for the control system and (ii) 2,2-diethoxyacetophenone (DEAP), not the dual enzyme system, was used as the initiator for Gel Vis under UV radiation. The resulting control system was therefore a viscous solution. These were selected in comparison with free GOx/HRP and immobilized GOx/HRP in Gel II. According to Fig. 5a, it is interesting that the initial reaction rate of the immobilized GOx/HRP (Gel II) at 5 mM OPD and 5 mM glucose is larger than the other systems in phosphate buffer (pH = 7.0).

As shown in Table S1,† the kinetic parameters of GOx/HRP are calculated from the Lineweaver–Burk plots of the initial rates at different concentrations (Fig. S5†). The immobilized GOx/HRP in Gel II reveals superactivity, as indicated by the highest *k*_cat_ value (turnover number, 7.348 s^–1^), which is approximately 1.16 ± 0.05 times, 1.29 ± 0.06 times and 1.31 ± 0.06 times higher than those of free GOx/HRP, the control system and the immobilized GOx/HRP in Gel Vis, respectively. The activity of the control system is lower than the free GOx/HRP group due to the partial loss of activity during the enzyme-initiated reaction of PEGMA. Therefore, the original activity of the immobilized GOx/HRP in Gel II is 1.26 ± 0.05 times (calculated according to values in Table S2†) higher than the equivalent free GOx/HRP. The superactivity of the immobilized GOx/HRP in Gel II might be similar to consecutive reactions within subcellular organelles of eukaryotic cells, which minimizes the diffusion of intermediates and thus boosts the overall reaction efficiency.^35^,^36^

**Fig. 6:**
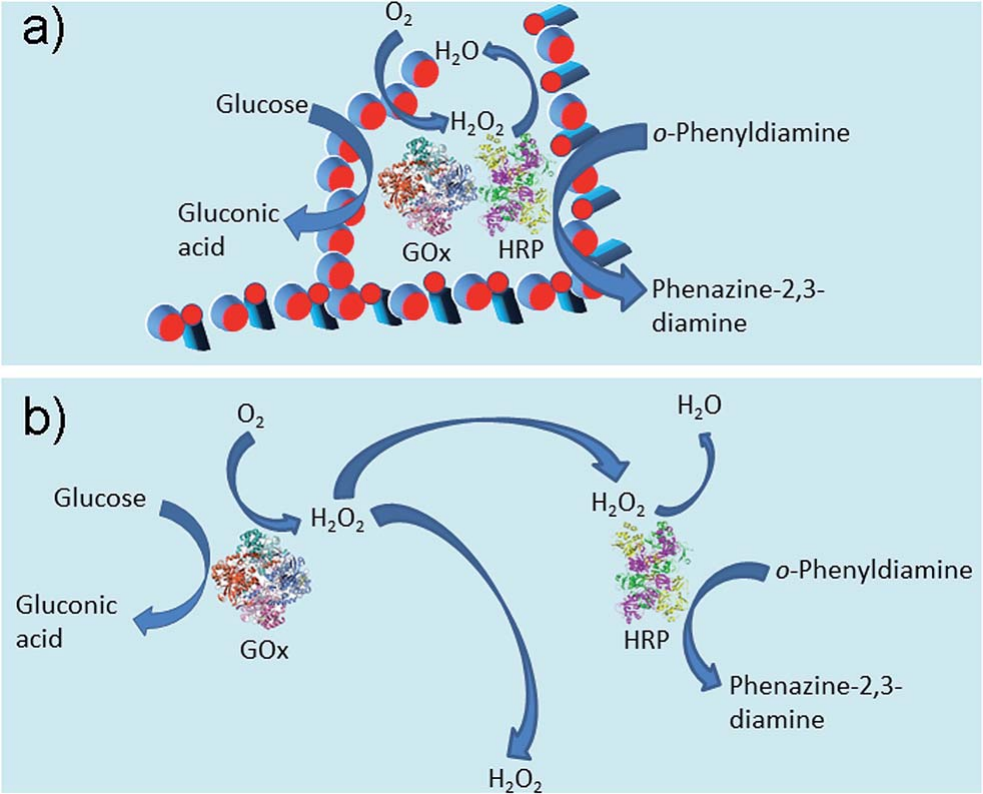
Enzymatic reaction illustrations of immobilized enzymes in Gel II (a) and free enzymes (b).

In our system, as shown in Fig. 1c, the immobilized dual enzyme might co-localize closely in the matrix of Gel II during the process of polymerization/cross-linking. When the enzymatic reactions are conducted, GOx initially catalyzes the oxidation of glucose to gluconic acid with the formation of H_2_O_2_. Furthermore, the latter product promptly participates in the HRP-mediated oxidation of OPD to generate the coloured product, phenazine-2,3-diamine, *λ* = 450 nm (Fig. 6a). It should be noted that in the other system (with the two free enzymes), the enzymes are separated by larger distances, and the generated H_2_O_2_ partially diffuses into the bulk solution, thus resulting in a lower local concentration of H_2_O_2_ around the HRP (Fig. 6b). Therefore, the superactivity might be mainly ascribed to the following three factors: (i) GOx and HRP are closely immobilized into the matrix of Gel II during the polymerization/cross-linking process, (ii) the generated H_2_O_2_ is utilized instantly by neighbouring HRP before diffusion to the bulk solution, and (iii) the porous network facilitates mass transport.^37^,^38^ Because the dual enzyme is closely localized in the porous Gel II, the *K*_m_ value for the immobilized GOx/HRP in Gel II is lower than the immobilized GOx/HRP in both Gel Vis and the controls.

Furthermore, the immobilized dual enzyme, GOx/HRP, exhibited reusability and thermal stability. As shown in Fig. S6,† GOx/HRP in Gel II maintained 53% of its initial enzymatic activity after incubation in water at 60 °C for 50 min, whereas the free enzymes maintained 0.5% of their initial activity after 50 min incubation. Thus, the hybrid gel maintains the enzyme activity in the early stage. The thermal stability might be ascribed to the fact^37^ that enzymes were immobilized in the gel matrix *via* strong non-covalent interactions between the enzymes and nanofibers; therefore, the stereo-configuration of the dual-enzyme might be well maintained when they were incubating at 60 °C and this results in enhanced thermal stability compared to the free enzymes. We compared the fresh and recovered Gel II during 15 min oxidation of OPD in buffer. Demonstrating its reusability, the immobilized enzymes maintained 72% of their initial activity after five catalytic cycles (Fig. S7†). Therefore, the hydrogel is an ideal scaffold for enzymes.

Then, degradability was investigated.^39^ The digestion result (Fig. 5b) illustrates that the hydrogelators were easily hydrolysed by proteinase K and only 29% of NapFFK-acrylic acid remained in solution after 24 h incubation. Furthermore, Gel II containing Congo red was incubated with proteinase K, which indicates that the amount of proteinase K could affect the release rate of Congo red encapsulated in Gel II (Fig. S8†). The cell release experiment was also conducted *via* the Alamar blue cell viability assay. As shown in Fig. S9,† 25% cells were released from Gel II after culturing with proteinase K for 24 h. This is mainly ascribed to the fact that the decomposition of the hydrogelators resulted in the destruction of the supramolecular networks, and this further broke the regular arrangement of the hybrid hydrogel frameworks. The degradable hydrogel is a new candidate for tissue engineering.

Based on its porous structure and the comfortable media for enzymatic reactions, the hybrid hydrogel Gel II was used in cell printing to construct 3D structures of cells (Fig. 4c). Prior to 3D bioprinting, Gel II was used to *in situ* 3D cell culture and NIH-3T3 cells were chosen as the model cell line. In a typical process, NIH-3T3 cells were added to the precursor solution of Gel II and mixed thoroughly. The precursor solution can maintain the cells in a suspension due to its viscosity, thus preventing cell settlement and aggregation, which facilitates cell printing. After culturing for 48 h, the cells were stained with fluorescein diacetate (FDA) and propidium iodide (PI), and they maintained high viability at 98.5% ± 1.8% (Fig. S10†). This result elucidates that the hybrid hydrogel is non-toxic to cells and permeable for nutrients, which are promising properties for a bio-scaffold.^40^,^41^ Furthermore, the hybrid hydrogel was used in cell printing. Fig. 4d shows a 3D printed stack of NIH-3T3 cells in the hybrid hydrogel stained with FDA and PI. A live/dead assay gave a viability of 99.2% ± 1.5%.

## Conclusions

In summary, we have developed a new strategy utilizing dual enzyme-initiated cross-linking polymerization to prepare a tough supramolecular-polymeric hybrid hydrogel, which could be used for *in situ* 3D cell printing to construct designed 3D structures. Co-immobilized GOx/HRP during gelation exhibited superactivity in buffer compared to free enzymes due to the synergistic effect of GOx and HRP in close proximity. In addition the hybrid hydrogel exhibited thermal stability and reusability. Furthermore, the cytocompatible hybrid hydrogel can be used for *in situ* 3D cell culture. Overall, the biodegradable hydrogel is a promising scaffold for biocatalysis and tissue engineering.

## Supplementary Material

Supplementary information
