# Supplementary material for: Printable hybrid hydrogel by dual enzymatic polymerization with superactivity
Source: Chem Sci. 2016 Jan 4;7(4):2748–52. doi: 10.1039/c5sc02234g (PMC5477016; doi:10.1039/c5sc02234g)
Supplement: Supplementary file 1 [file SC-007-C5SC02234G-s001.pdf]

Electronic Supplementary Material (ESI) for Chemical Science.
This journal is © The Royal Society of Chemistry 2016

Electronic Supplementary Information for

# Printable Hybrid Hydrogel by Dual Enzymatic Polymerization with Superactivity

Qingcong Wei,[a] Mengchi Xu,[b] Chuanan Liao,[a] Qing Wu,[a] Mingyu Liu,[c] Ye Zhang,*[d] Chengtie Wu,*[b] Liming Cheng,[e] and Qigang Wang*[a]

**Contents**

**Index** **Page**

1. **Materials……………………………………………………………S2**
2. **Hydrogelation of Gel I and Gel II………………………………………S2**
3. **Pre-treatments and characterizations……………………………………S3**
4. **NapFFK-acrylic acid conversion test……………………………………S4**
5. **Digestion test of NapFFK-acrylic acid…………………………………S5**
6. **Release of Congo red from Gel II………………………………………S5**
7. **Alamar blue assay………………………………………………………S5**
8. **3D cell culture……………………………………………………………S6**
9. **3D cell printing…………………………………………………………S6**
10. **Test of the catalytic activity……………………………………………S6**
11. **Test of the reusability…………………………………………………S7**
12. **Figures and Table………………………………………………………S8**
13. **References……………………………………………………………S14**

## 1. Materials

Horseradish peroxidase (HRP, EC 1.11.1.7, MW = 40Kda, 300 U/mg) was purchased from Shanghai Baoman Biotechnology Co., Ltd. Glucose oxidase from *Aspergillus niger* (GOx), Proteinase from *Engyodontium album* and poly (ethylene glycol) methacrylate (PEGMA, average MW = 360 g/mol) were purchased from Sigma-Aldrich. 2-[4-(2-hydroxyethyl)-1-piperazinyl]ethanesulfonic acid (HEPES, 1M) was purchased from Beijing Solarbio Science & Technology Co., Ltd. Congo red was purchased from Aladdin Industrial Inc. (Shanghai, China). Concentrated hydrochloric acid (HCl, 37.5 wt%), sodium hydroxide (NaOH), glucose and acetyl acetone (AcAc) were purchased from Sinopharm Chemical Reagent Co., Ltd. (Shanghai, China). All materials were used as received without further purification.

## 2. Hydrogelation of Gel I and Gel II

6-acrylamido-2(2-(2-(2-(naphthalene-2-yl)acetamido)-3-phenylpropanamido)-3-phenylpropanamido)hexanoic acid (NapFFK-acrylic acid) was synthesized according to the literature.[S1, S2] 2-(Naphthalen-2-yl)acetyl-(L)-Phe-(L)-Phe-(L)-Lys (NapFFK) was synthesized firstly via standard solid phase peptide synthesis (SPPS) on a 2-chlorotrityl chloride resin by successive coupling of Fmoc-protected-L-amino acids. And then NapFFK was reacted with acrylic acid using 1-Ethyl-3-(3-dimethylaminopropyl) carbodiimine (EDC) and N-hydroxysuccinimide (NHS) as coupling reagents to obtain the target product (Scheme S1a).

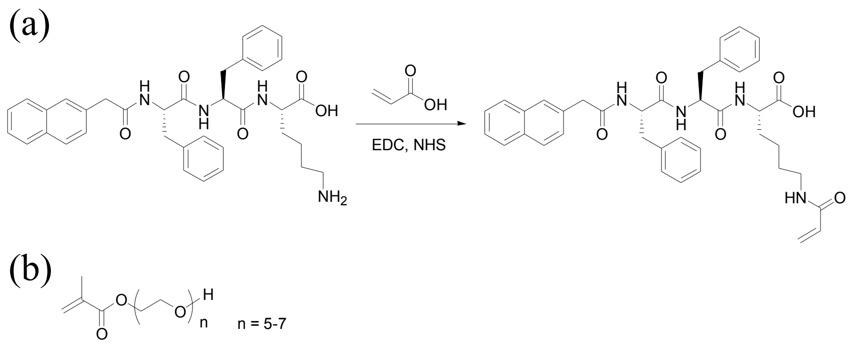


***Scheme S1***. (a) The synthesis reaction equation of NapFFK-acrylic acid. (b) General molecular formula of PEGMA.

*Preparation of Gel I:* NapFFK-acrylic acid (5.0 mg) and 1 N NaOH solution were added to 260 μL distilled water, and the mixture was sonicated for thorough dissolution. Then diluted HCl solution was added carefully until the pH reached 6-7, the volume was adjusted to 325 μL by adding water. After the mixture was mixed thoroughly on vortex, the colorless transparent solution was kept at 25 °C for 5 min and Gel I (1.54 wt% of NapFFK-acrylic acid) was formed. In addition, the supramolecular hydrogel used for rheological and compressive tests and other tests contains 0.96 wt% of NapFFK-acrylic acid (which is the minimal concentration to form the supramolecular hydrogel).

*Fabrication of Gel II:* The dual enzyme-catalyzed reagents including 50 μL $glucose_{(aq)}$ (40 mM), 10 μL $HRP_{(aq)}$ (42 mg/mL), 10 μL AcAc and 25 μL PEGMA were added to the Gel I (325 μL), and the mixture was mixed thoroughly on vortex. Finally, 100 μL $GOx_{(aq)}$ (10mg/mL) was added, after the yellow transparent precursor solution was mixed totally and kept at 37 °C for 35 min, Gel II (0.96 wt% of NapFFK-acrylic acid) was formed.

*Fabrication of Gel II containing Congo red:* The procedure was the same as Gel II except 0.5 mg Congo red was added to the precursor solution.

## 3. Pre-treatments and characterizations

*Pre-treatment of SEM samples:* The silicon wafer coated with samples was freezed in liquid nitrogen, and further dried 24 h in vacuum. The samples were sputter-coated wth a thin layer of gold before testing with a field emission scanning electron microscopy (Hitachi S-4800) at a 3 KV voltage.

*Pre-treatment of TEM samples:* The samples were coated on the carbon-coated copper grid and lyophilized. The pictures were acquired with a transmission electron microscopy (JEM-2010) at a 80 KV voltage.

*Rheological characterization:* The rheological properties of hydrogels were tested using a RS6000 rheometer (Thermo Scientific, Karlsruhe, Germany) with a parallel plate geometry (25 mm diameter, 0.3 mm gap) at 25°C. The frequency-dependent sweep was taken as a function of angular frequency at fixed strain of 0.03 %. The

strain amplitude sweep of gels was carried out at a fixed frequency of 1 Hz. The self-recovering process of Gel II in response to applied shear forces was performed using continuous step strain sweep test with alternate small oscillation force ($\gamma$ = 0.03 %) and large one ($\gamma$ = 50 %).

*Mechanical analysis:* The compressive test of hydrogels was taken on a FR-108B testing machine (Farui Co., China) at a crosshead speed of 1mm·min$^{-1}$. The diameter of the gels is about 13 mm and the thickness is 3-4 mm. The compressive stress ($\sigma$) was approximately calculated as $\sigma = F_{load}/\pi R^2$, where R is the original radius of the specimen. The compressive strain ($\varepsilon$) is defined as the change of the thickness relative to the original thickness. The stress and strain between $\varepsilon$ = 5 and 15 % were used to calculated the Young's modulus with at least parallel tests for each hydrogel.

*Electron Paramagnetic Resonance (EPR) measurement:* The EPR spectrum was recorded on a JEOL Spectrometer (JES FA200) operating at 9.50 GHz. In a typical process, the two solutions (solution 1 was mixed by 100 μL $GOx_{(aq)}$ and 160 μL $H_2O$, solution 2 was mixed by 50 μL $glucose_{(aq)}$, 10 μL $HRP_{(aq)}$, 10μL AcAc and 190 μL $H_2O$) were mixed with the same volume, and then the mixture was rapidly transferred to a standard capillary and placed into the EPR spectrometer, and POBN was used as the spin trap. The spectrum was recorded after 5 min reaction.

*3D printing:* The precursor solution of Gel II was loaded into the 3D printer (Nano-Plotter NP 2.1, GeSiM, Grosserkmannsdorf, Germany) of Shanghai Institute of Ceramics (Chinese Academy of Sciences). Different arrays and patterns were designed and printed.

## 4. NapFFK-acrylic acid conversion test

In a typical control test, 5 mg NapFFK-acrylic acid was dissolved in $d_6$-DMSO with acetonitrile as internal standard substance. Then Gel II (containing 5 mg NapFFK-acrylic acid in the precursor solution) was crushed and dispersed in NaOH(aq) (pH = 10). The resulting mixture was stirred violently for 2 hours and filtered. The filtrate was centrifuged at 10000 rpm for 20 min and the supernatant was acidized by 1M HCl solution to pH 2-3. The white precipitate was obtained by

centrifuging and dried, then it was dissolved in $d_6$-DMSO with the same amount of acetonitrile as internal standard substance compared to the control. Three parallel experiments were taken and the results were presented as mean values ± Standard Deviation. The conversion ratio of gelators was 75.3 % ± 2.8 %.'

## 5. Digestion test of NapFFK-acrylic acid

1.25 mg NapFFK-acrylic acid was added to 6 mL (0.167 M) HEPES buffer containing 120 $\mu$L (150 U/mL) proteinase K. The solution was incubated at 37 °C and 20 $\mu$L of solution was taken out each time to test the amount of NapFFK-acrylic acid remained by HPLC.

## 6. Release of Congo red from Gel II

Four groups of HEPES buffer solution (4 mL, 0.167 M) containing 0.26 mL Gel II (incorporating 0.25 mg Congo red) were treated with different concentrations of proteinase K, 0 U/mL, 3 U/mL, 6 U/mL and 12 U/mL respectively. The solutions were incubated at 37 °C, and the intensity of UV absorbance of Congo red (488 nm) was measured at different times.

## 7. Alamar blue assay

NIH-3T3 cells were added to the precursor solution (500 μL) of Gel II and mixed thoroughly, then the mixture was transferred to 24-well tissue culture plates at a density of 20,000 cells per well with 500 μL of fresh culture medium. Then proteinase K (48 U in 100 μL culture medium) was added to the experimental group (defined as E-group) while 100 μL culture medium was added to the control group (defined as C-group), and the two groups of cells were cultured for 24 h at 37°C. The culture medium was aspirated and the gels were washed with 500 μL of 0.1 M PBS solution. Then 300 μL of fresh medium containing a 10 % solution of Alamar blue to the wells and keep culturing for another 3 hours. Finally 150 μL medium with dye alone was transferred to a 96-well plate and light absorbance at 600 nm was recorded. And three parallel tests were taken for each group.

## 8. 3D cell culture

NIH-3T3 cells ($1 \times 10^6$ cells $mL^{-1}$, 50 μL) were added to the precursor solution (0.96 wt% of NapFFK-acrylic acid, 520 μL) of Gel II in a vial and mixed thoroughly. The resulting mixture was incubated at 37 °C for 30 min in terms of gelation, and then the Gel II was transferred to serum-free Dulbecco's Modified Eagle's Medium (DMEM) containing 10 % (v/v) of foetal bovine serum and 1 % (v/v) penicillin-streptomycin in a glass coverslip. After culturing for 48 h, the cells were stained with 40 μg/mL fluorescein diacetate (FDA) and 10 μg/mL propidium iodide (PI) in DMEM media and incubated at 37 °C for 35 min. Images were acquired using a Leica TCS SP8 confocal laser scanning microscope, and illumination was provided by an argon gas laser at 488 nm or a 561 nm diode laser. 3D stacks were acquired with a z-step size of 4 μm. A live/dead staining assay was used to calculate the viability of the cells. And the viability was calculated via the Imaris Spot Detection function in order to determine the relative proportion of live (green, FDA stained) or dead (red, PI stained) cells in different areas of view. The data are presented as mean values ± Standard Deviation. Figure S10 showed that the cultured cells were encapsulated and distributed in the Gel II in 3D. And the viability of live/dead assays was 98.5 ± 1.8 %.

## 9. 3D cell printing

3T3 cells ($1 \times 10^6$ cells $mL^{-1}$, 50 μL) were added to the precursor solution (0.96 wt% of NapFFK-acrylic acid, 520 μL) of Gel II and mixed thoroughly, then the mixture was loaded into the printer. Grids were printed with 3 layers. The printed grids were incubated at 37 °C for 35 min in terms of gelation. And further, the grids were stained with 40 μg/mL FDA and 10 μg/mL PI in DMEM media and incubated at 37 °C for 30 min, and imaged using a Leica TCS SP8 confocal laser scanning microscope.

## 10. Test of the catalytic activity

The oxidation of *o*-phenylenediamine (OPD) by $H_2O_2$ was selected as a model reaction with glucose and OPD as substrates to test he catalytic activity of free dual

enzymes and immobilized dual enzymes. Typically, 10 μg GOx and 4.2 μg HRP (or a piece of Gel II containing 10 μg GOx and 4.2 μg HRP) were added into a solution containing OPD (fixed 10 mM) and glucose (a series of concentrations 10 mM, 7.5 mM, 5 mM, 3 mM, 2 mM and 1 mM) in buffer (PBS, pH = 7.0) for catalyzing the reaction..

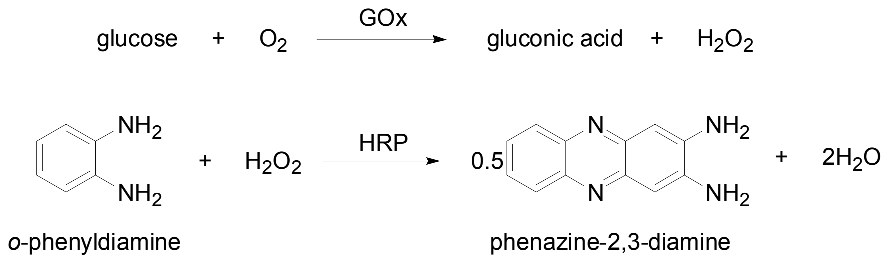


The increase in absorbance at 450 nm for the oxidation product (phenazine-2,3-diamine) was measured by a UV-Vis spectrometer (UV-2700, Shimadzu) at 0.1-min intervals. The concentrations of the colorful product were corrected according to the molar extinction coefficient ($\varepsilon_{450}$ = 16300 $M^{-1}\cdot cm^{-1}$) in aqueous buffer. Then the initial reaction rate was obtained by linear fitting the product concentration with time. We obtained the Lineweaver-Burk plot according to a series of initial reaction rates to estimate the kinetic constant values. The typical characterization of enzymatic reaction is the Michaelis-Menten equation $V_0 = V_{max}\cdot[S]/([S] + K_m)$, including the maximum reaction rate $V_{max}$, Michaeliis-Menten constant $K_m$, and turnover number $k_{cat} = V_{max}/[Enzyme]$, can represent the activity of the enzyme.

## 11. Test of the reusability

The fresh and recovered Gel II were used to test the reusability via the oxidation of OPD (10 mM) at a fixed glucose concentration (3 mM) in 2 mL aqueous buffer. The total amount of phenazine-2,3-diamine was measured by UV-Vis spectrometer after 15 min reaction. The recovered Gel II was washed with ethanol and buffer, respectively in order to remove the product. Then the recycled Gel II was mixed with a fresh reaction mixture again to catalyze the reaction.

## 12. Figures and Tables

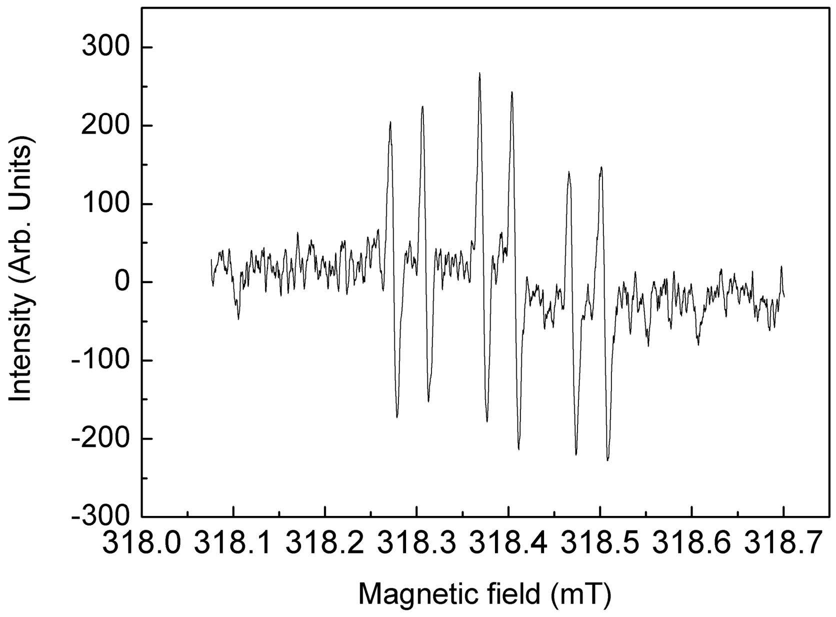


***Figure S1.*** EPR spectra of the POBN radical adducts formed in the dual enzyme-mediated redox initiation system without the addition of PEGMA.

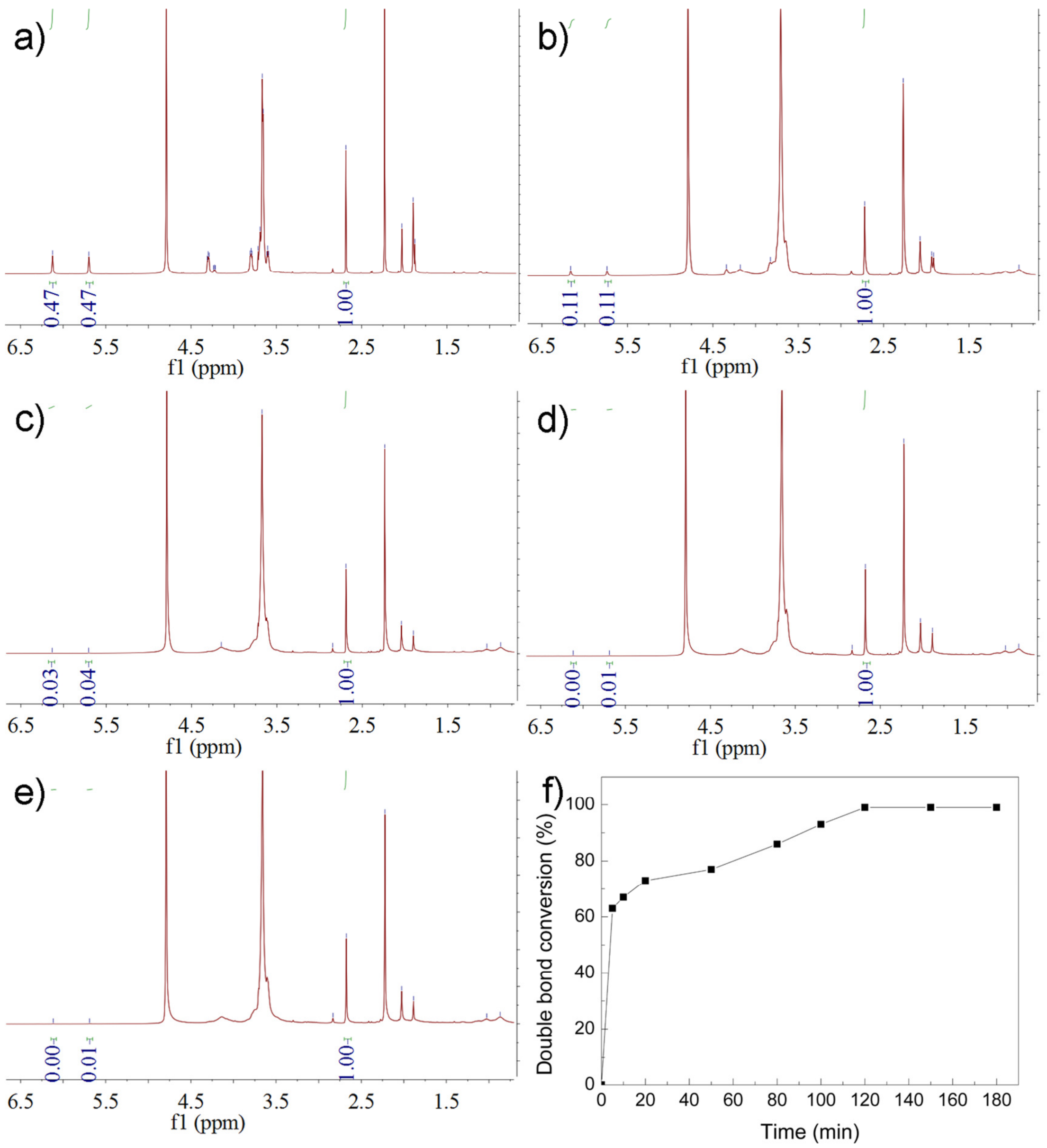


***Figure S2.*** The $^{1}$H NMR spectra of a $D_2O$-substituted precursor (the high concentration supramolecular hydrogel with the dual enzyme-catalyzed system) at different reaction times: 0 min (a); 50 min (b); 100 min (c); 120 min (d); 180 min (e). The conversion of vinyl double bonds (PEGMA) in the Gel II is calculated using dimethyl sulfoxide as an internal standard (f).

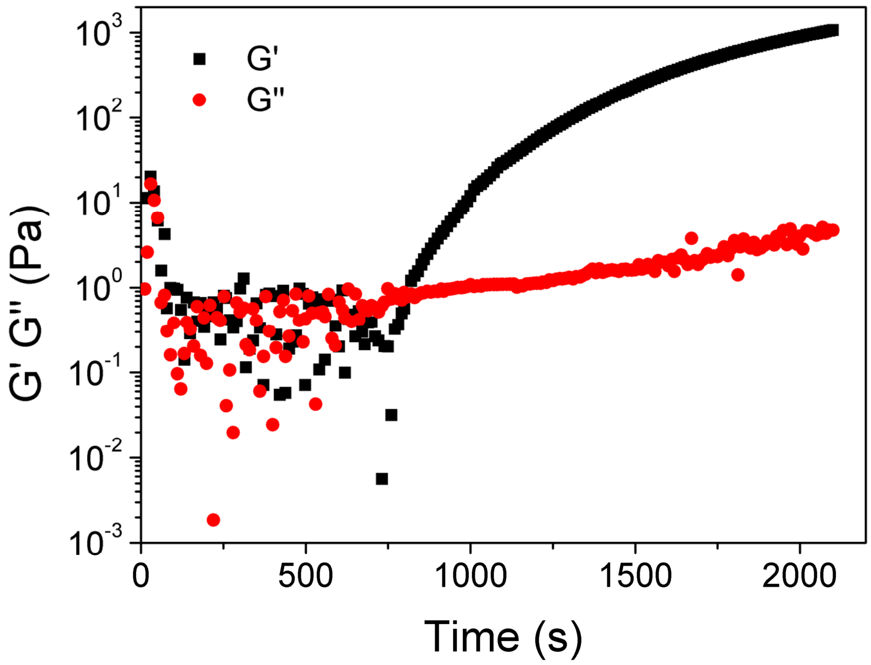


***Figure S3.*** Dynamic time sweep measurement at a constant strain of 0.03 % and a constant frequency of 1 Hz.

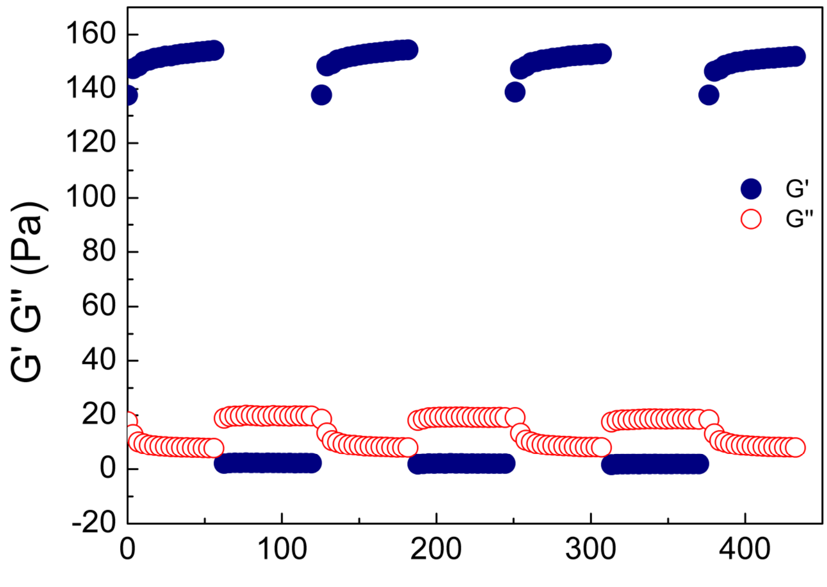


***Figure S4.*** The self-recovering nature of Gel II after large amplitude oscillatory breakdown.

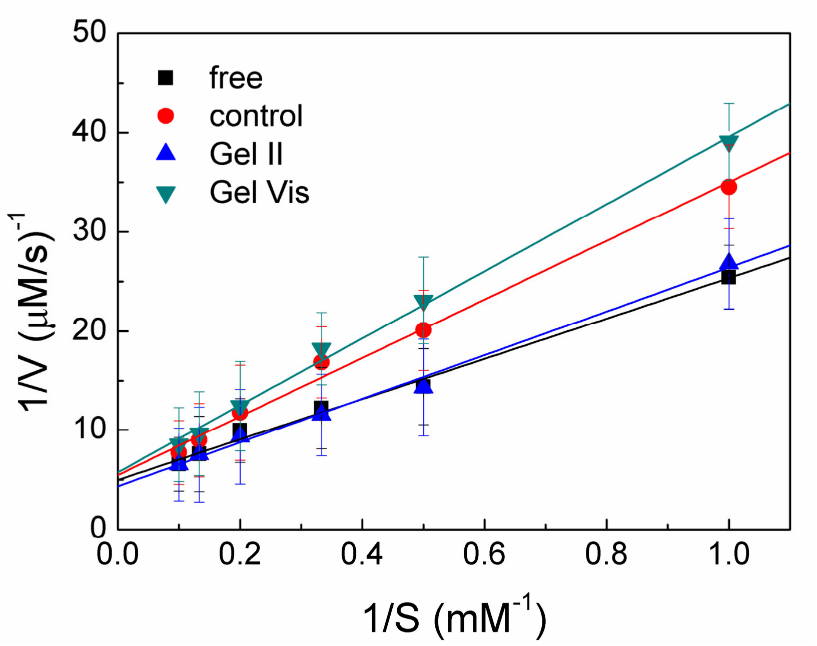


***Figure S5.*** Lineweaver-Burk plots of four systems in buffer (PBS, pH = 7.0).

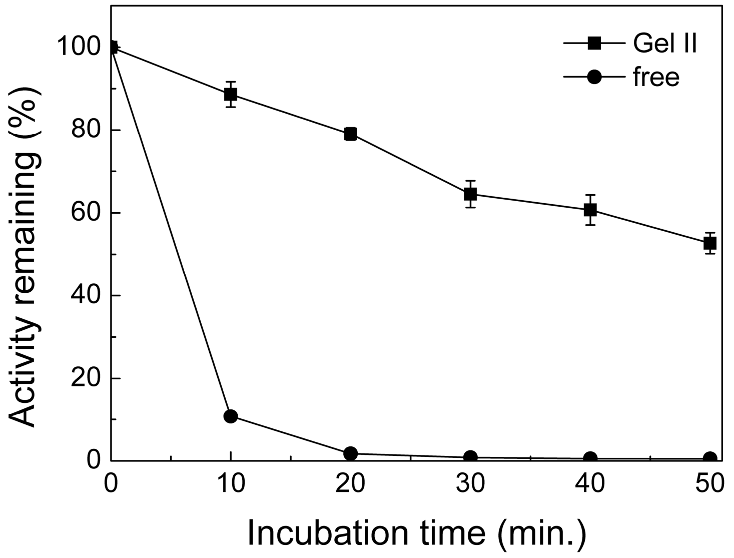


***Figure S6.*** The remaining activity of free enzymes and immobilized enzymes in Gel II in buffer after incubation at 60 °C for different periods of time.

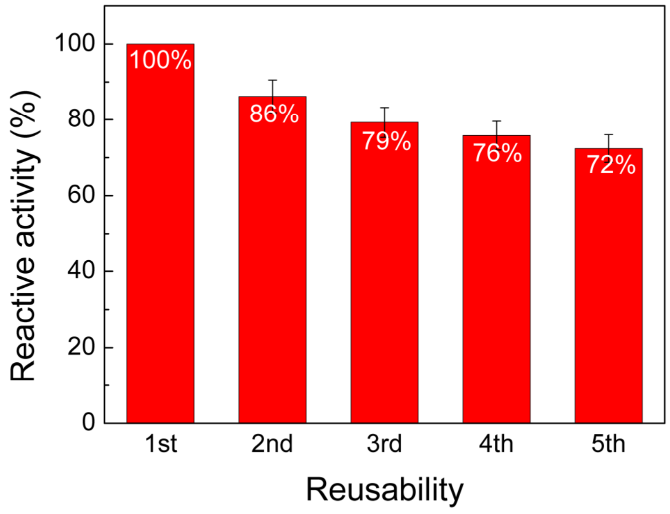


***Figure S7.*** The reusability of Gel II in buffer (PBS, pH = 7.0).

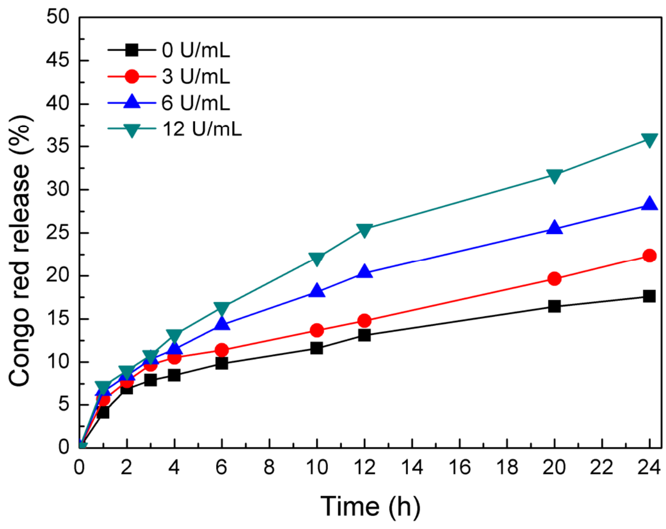


***Figure S8.*** Controlled release of Congo red from Gel II by proteinase K.

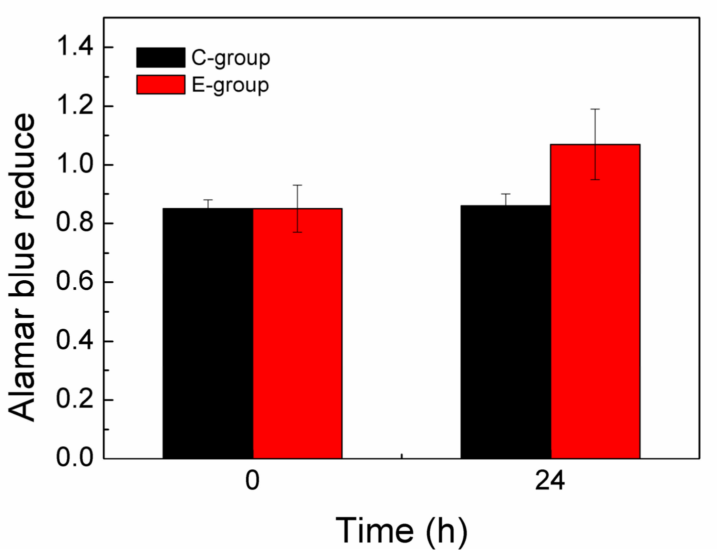


***Figure S9.*** The cell release test of Gel II via Alamar blue assay.

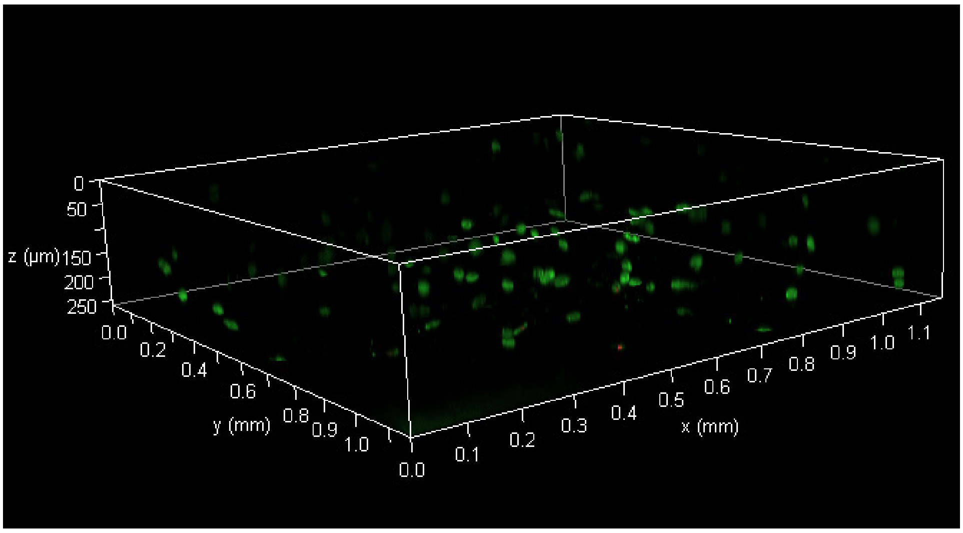


***Figure S10.*** A 3D stack of 3T3 cells in the hybrid hydrogel Gel II with FDA and PI staining and imaged after 48 h culture (gridlines for x and y axis: 0.1 mm; gridlines for z axis: 50 μm).

Table S1. The modulus values of a series of hybrid hydrogels

| Hybrid hydrogels | NapFFK-acrylic acid (mg) | PEGMA (μL) | G' (Pa) | G" (Pa) |
|---|---|---|---|---|
| 1 | 5 | 10 | 574 ± 34 | 31.5 ± 7.5 |
| 2 | 5 | 25 | 1030 ± 35 | 60 ± 5 |
| 3 | 5 | 50 | 1061 ± 68 | 72.5 ± 12.5 |
| 4 | 5 | 75 | 1264 ± 62 | 135 ± 41 |
| 5 | 5 | 100 | 1298 ± 40.5 | 81 ± 14 |

Table S2. Reaction kinetic constants of different GOx/HRP systems.

| | $V_{max}$ ($\mu$M/s) | $k_{cat}$ ($s^{-1}$) | $K_m$ (mM) |
|---|---|---|---|
| Free | 0.198 | 6.340 | 4.010 |
| Control | 0.178 | 5.71 | 5.223 |
| Gel II | 0.230 | 7.348 | 5.054 |
| Gel Vis | 0.175 | 5.610 | 5.938 |

**13. References**

S1. Y. Zhang, N. Li, J. Delgado, Y. Gao, Y. Gao, Y. Kuang, S. Fraden, I. R. Epstein, B. Xu. *Langmuir* **2012**, *28*, 3063-3066.

S2. Y. Zhang, R. Zhou, J. Shi, N. Zhou, I. R. Epstein, B. Xu. *J. Phys. Chem. B* **2013**, *117*, 6566-6573.
